# A Unique Case of Late Presentation Giant Lower Extremity Malignant Melanoma

**DOI:** 10.7759/cureus.55414

**Published:** 2024-03-02

**Authors:** Omar Al Zarkali, Amanda Marker, Lakhvir Kaur, Ana VanDerWall

**Affiliations:** 1 Internal Medicine, HCA Florida Blake Hospital, Bradenton, USA; 2 Medical Oncology, HCA Florida Blake Hospital, Bradenton, USA

**Keywords:** cutaneous malignancy, oncology imaging, cancer immunotherapy, giant melanoma, malignant melanoma metastasis

## Abstract

This case describes a unique presentation of a rare malignancy: giant melanoma. Due to the accessibility of healthcare in the United States, it is unusual for melanomas to grow to massive sizes without clinical intervention. In fact, an in-depth literature review elicited only a handful of similar cases. Giant malignant melanomas are typically defined by a cutoff size of no less than 10 cm in diameter. They often present with distant metastases and are highly invasive. Due to limited yet highly variable presentations, there is no standardized approach to treating this class of melanomas. We present a case with unique features not previously documented in similar cases that was ultimately treated with a novel approach.

## Introduction

Giant melanomas are a rare subtype of malignant melanomas defined as lesions measuring greater than 10 cm in diameter [1]. These are uncommon not only for their rare incidence in general but also because patients typically seek medical attention prior to these tumors reaching such a large size. With the exception of one case report in which a 14.5 x 10.4 cm mass grew within three months, most of these lesions grow over six months prior to reaching such massive sizes [2]. Giant melanoma patients are typically aware of their disease course and are even able to describe the initial onset and progression of their lesions due to their superficial etiology. Unfortunately, at the time of the patients' presentations, their lesions are often found to have already metastasized, as was the case with our patient. Upon his first encounter for evaluation of his lesion, he was found to have numerous metastases to his lungs as well as a suspicious distant lesion on his left arm.

## Case presentation

A 63-year-old Caucasian male presented with an enlarging, painful left lower extremity mass of one-year duration. His late presentation was secondary to apprehension surrounding the COVID-19 pandemic. Associated symptoms included night sweats, fatigue, palpitations, anorexia, abdominal distention, and lower extremity edema. He also noted another mass on his left upper extremity that has been progressively growing in a similar fashion to the original mass, however, was much smaller and only became noticeable a few months ago.

**Figure 1:**
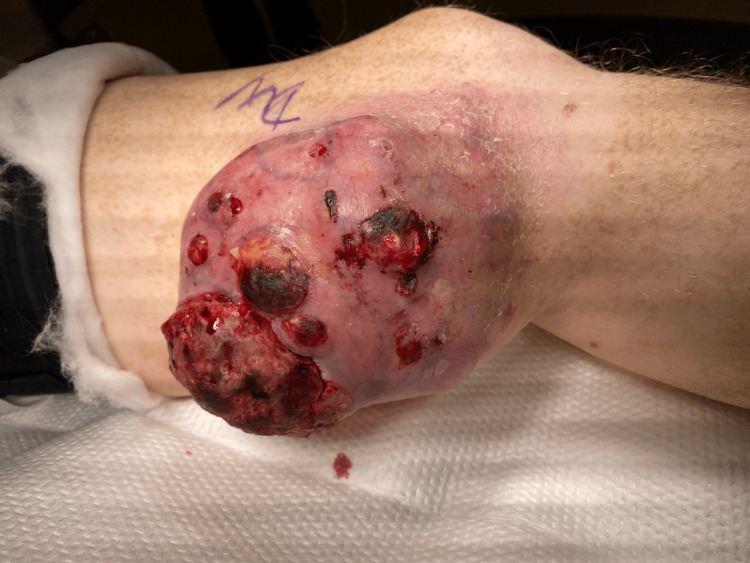
Giant melanoma at the time of presentation

**Figure 2:**
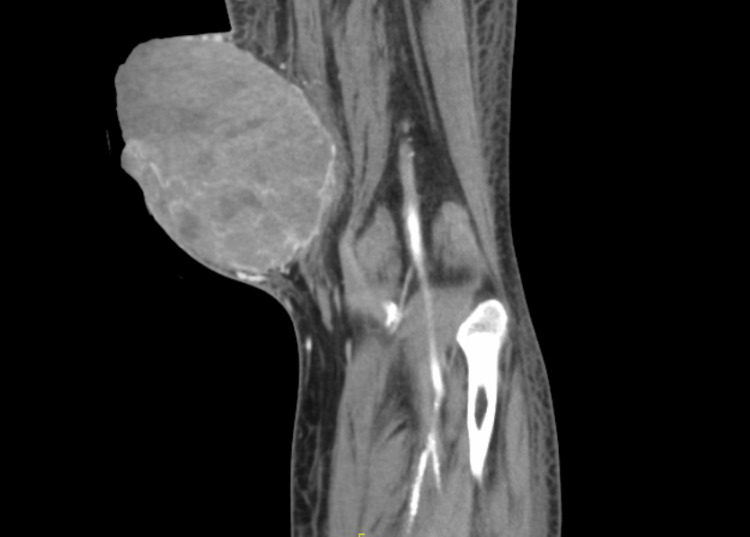
Computed tomography angiography (CTA) of the left lower leg

**Figure 3:**
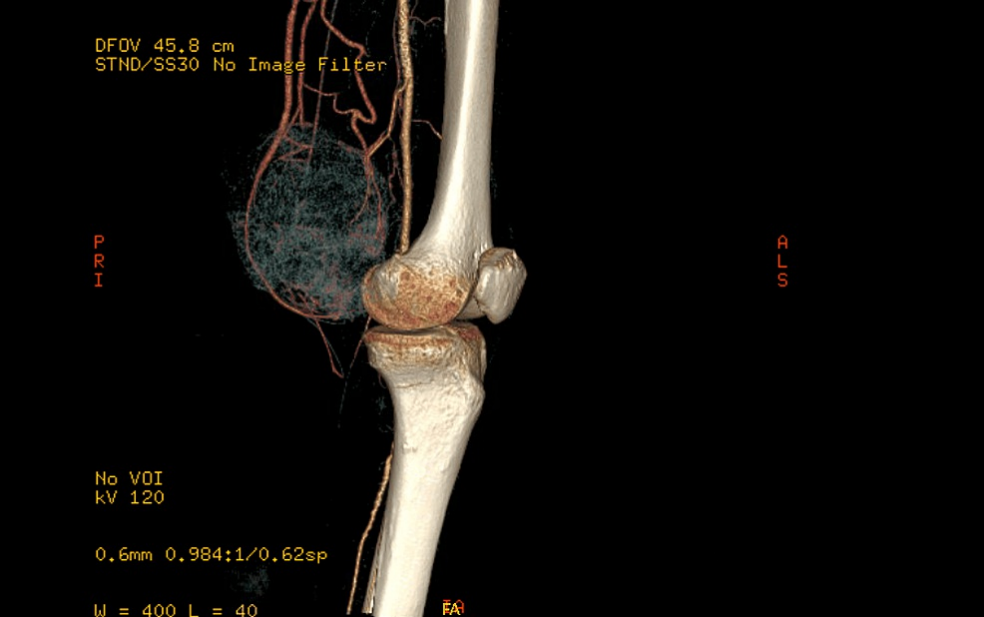
Computed tomography angiography (CTA) of the left leg with 3D reconstruction

**Figure 4:**
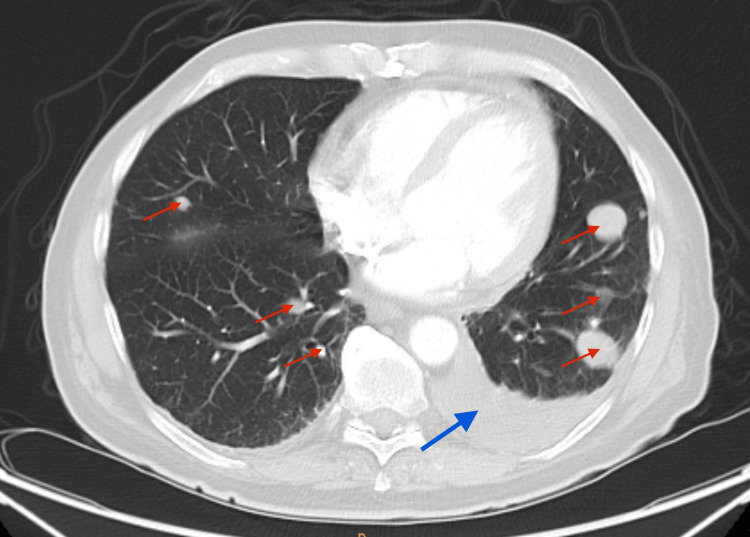
Computed tomography angiography (CTA) of the chest. Multiple bilateral pulmonary nodules (red arrows) and large left pleural effusion (blue arrow)

On physical examination, there was a large, pigmented, friable, ulcerated, vegetative mass approximately 10 cm in diameter on the left distal medial thigh with multiple ulcerations (Figure 1). A left lower extremity ultrasound revealed a large irregular-echogenicity and multilobulated mass measuring 9.6 x 10.2 x 10.8 cm as well as thrombi of left superficial femoral, popliteal, and posterior tibial veins. CT with contrast of the left leg demonstrated a large mass with no evidence of invasion of nearby bones (Figures 2, 3). The chest x-ray revealed multiple lung nodules consistent with metastatic disease, and a computed tomography angiography (CTA) of the chest demonstrated a left pleural effusion as well as innumerable well-circumscribed lung nodules (Figure 4). The CTA of the chest was repeated and demonstrated thrombi in the distal arterial branches of the left lower lobe with multiple pulmonary metastases measuring up to 2.5 cm on the right and 2.69 cm on the left. CT abdomen and pelvis with contrast showed splenomegaly and mild ascites. An excisional biopsy was performed, which revealed BRAF V600E+ malignant melanoma on pathology (Appendix). The patient was treated medically and was discharged with close outpatient follow-up with oncology.

In the outpatient setting, he underwent a whole-body fluorodeoxyglucose-18 positron emission tomography (FDG-PET) scan, which revealed numerous bilateral pulmonary nodules that had increased in size compared to the prior CT of the chest, as well as new multiple hepatic metastases in both lobes of the liver. The PET scan also demonstrated an interval increase in the size of the left external iliac lymph node and left inguinal lymph nodes compared to prior CT scans. There were new osseous metastases within the proximal left femur and T2 vertebral body. The previously documented left upper extremity mass showed increased FDG activity as well. Splenomegaly, ascites, and pleural effusions mildly improved; however, a small pericardial effusion had worsened in the imaging studies. Brain MRI, bone marrow biopsy, and peripheral blood smear were unremarkable.

He was started on pembrolizumab and tolerated three courses of the immunotherapy drug. A repeat CT of the abdomen and pelvis at that time showed an increase in the size of the hepatic mass along the anterolateral aspect of the dome of the liver, along with a new adjacent necrotic peritoneal metastasis in the right paracolic gutter. The scan also showed a new subcutaneous nodule in the left anterior pelvic wall; however, the osseous metastases remained stable. There was also improvement in the remaining adenopathy and metastases described in the initial PET scan. The patient then received two additional courses of pembrolizumab for a total of five at the time of submission for publication. Given the patient’s worsening functional status (the Eastern Cooperative Oncology Group (ECOG) score declined from one to four since initiating therapy) and mixed results with pembrolizumab, there was consideration of halting treatment and deferring to hospice. At the time of submission, a repeat PET scan was ordered prior to making a decision to give a sixth course of the immunotherapy drug.

## Discussion

An in-depth review of similar cases was done upon initial diagnosis of this patient revealing multiple unique factors related to this case not previously documented (Table 1).

**Table 1:** Review of similar cases LN, lymph node; TT, targeted therapy with dabrafenib (BRAF-i) and trametinib (MEK-i); CMO, comfort measures only; CT, chemotherapy.

Location	Size	IHC	Staging	Metastases/positive lymph node	Tx	Result
Right forearm [3]	14 × 7 × 12 cm	S100 (+) Melan-A (+)	T4bN0M0 Clark 5 Breslow 70 mm	NA	Surgery	Remission 4 years later
Antero-medial aspect of left arm [4]	9 × 10 × 15 cm	S100 (+) Melan-A BRAF (+)	Stage IV Clark 5 Breslow >10 mm	Lung/left axillary LN	Surgery, TT	Death: brain metastasis
Lateral left arm [5]	19 × 16 cm	S100 (+) Vimentin (+) SOX10 (+) V600E BRAF (+)	Stage IV	Left upper lung, left iliac wing	Surgery, TT	Remission 1 year later
Back [6]	8 × 6 cm	N/A	T4N2M1c Breslow 48 mm	Back x2 (in-transit metastases)/bilateral axillary LN	Surgery	Death
Left upper arm [7]	10 × 8 × 3 cm	N/A	Breslow 31 mm Clark 5	Left axillary LN	Surgery	New metastases on vertebral column and ribs
Right upper arm [7]	23 × 21 × 6 cm	S100 (+) HMB45 (+) tyrosinase (+)	Breslow 75 mm Clark 5	Brain, right upper arm (in-transit)/right axillary LN	Surgery	Lost to follow-up
Anterior chest wall [8]	15 × 13 × 2.5 cm	BRAF (–) V600E (–)	Stage IV	Brain, lungs, liver, L4/retroperitoneal, and perisplenic LN	CMO	Death 15 months later
Left gluteal region [9]	18 × 16 × 5 cm	S100 (+) HMB45 (+) Ki67 (+)	Stage IV Clark 5 Breslow 48 mm	Lung/left inguinal LN	Surgery, declined further treatment	ICU 2 months later
Scalp [10]	12 × 10 cm	HMB45 (+)	Stage IV	Brain, spleen, liver/hilar LN	Plan for: surgery, CT, possible TT	N/A
Left shoulder [11]	20 × 20 cm	NRAS Q61L (+) S100 (+) Cyclin D1 (+) SOX 10 (+) MTF (+) HMB45 (+) MART1 (+)	T4N0M0 Clark 5 Breslow 70 mm	None	Surgery	Death from unknown cause; however, no recurrence 6 months later
Scalp [12]	14.5 × 10.4 cm	N/A	T4bN3M1 Clark 5 Breslow 18 mm	Left parotid gland, left ear cartilage/left axillary LN	Surgery	Poor prognosis
Lower back [13]	22 × 25 × 7 cm	S-100 (+) MART-1 (–) HMB-45 (rarely +)	Stage IV	None/left inguinal LN	Palliative surgery, CT	Disease regression

Unique to our patient is the presentation of a lesion on the lower extremity. During our research into prior similar cases, we were unable to find any cases that presented lower than the buttocks. Also unique to this case was the finding of a positive BRAF V600E mutation. BRAF mutations, while common in melanomas, were only found in two other cases of giant melanomas [4,5]. This genetic mutation, in a patient naive to systemic therapy, the patient’s functional status, and the metastatic nature of presentation, made treating with pembrolizumab an appropriate initial therapeutic option [14-16]. The use of this immunotherapy was not seen in other cases of similar melanomas. After multiple courses of pembrolizumab, a variable response was achieved throughout the different lesions. These results warrant further investigation into pembrolizumab in the treatment of similar malignancies in the future.

Another unique aspect of this presentation was the presence of a second, presumably soft tissue malignancy on the left upper arm. It is unclear at this time whether this lesion is a metastasis of the primary lesion on the thigh or a second independent primary cancer. While it is less likely for a patient to have two primary tumors, giant melanomas have not previously been documented to spread distally to soft tissue. Melanomas, in general, have a known history of spreading to soft tissue through various methods. These metastases can be local (satellite lesions), regional (in-transit), or distant lesions typically seen on imaging as they are easily differentiated from local subcutaneous adipose tissue [2]. Satellite lesions are defined as secondary lesions in the skin or subcutaneous tissue within 2 cm of the primary tumor [17]. Satellite lesions are clearly separated by the normal dermis and are considered intralymphatic extensions of the primary mass. In-transit lesions are more distant, however still within the regional nodal basin. Any soft tissue lesions beyond these limitations are defined as distant metastasis. In the case of our patient, given the lesions in question are on a lower and upper extremity, it is safe to say that it can be classified as a distant metastasis if not a second primary lesion. Only two other cases of giant melanoma in our literature review demonstrated soft tissue metastasis. The first patient presented with a giant melanoma on the back with two lesions later described as in-transit lesions [6]. The second patient had a primary tumor in the right upper extremity that was surgically resected with a split-thickness skin graft [7]. Two months later, he was found to have in-transit metastases at the graft site and upper arm. Whether our patient’s second lesion turns out to be a distant metastasis or a second primary tumor, it remains a previously unseen feature of giant melanomas.

## Conclusions

After appropriate work-up and treatment of our patient, as well as completing a comprehensive literature review, we conclude that the vast array of presentations of giant melanomas makes it challenging to devise a standardized treatment approach. Treatment modalities utilized to treat these patients varied, and while not all were successful, they were not incorrect. It is important to treat these patients uniquely in order to target therapy to their individual presentations. All aspects of the disease, including pathology, staging, sites of metastases, comorbidities, and functional status, should be taken into consideration when devising a multimodal approach to treat patients with giant malignant melanomas. Further investigation into similar cases is necessary to gain deeper knowledge of this rare and unique cancer.

## Figures and Tables

**Figure 5:**
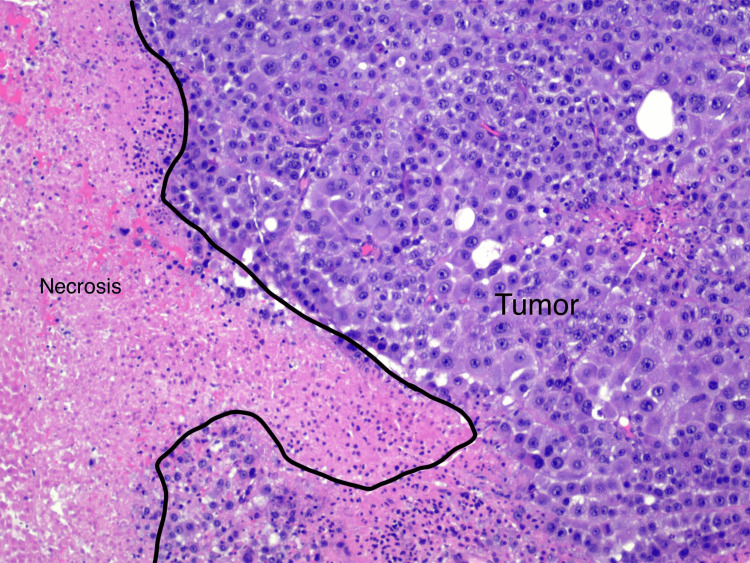
Hematoxylin and eosin stain (H&E), 10x. Sheets of large pleomorphic cells and necrosis. Cells have abundant cytoplasm, large nuclei with prominent nucleoli

**Figure 6:**
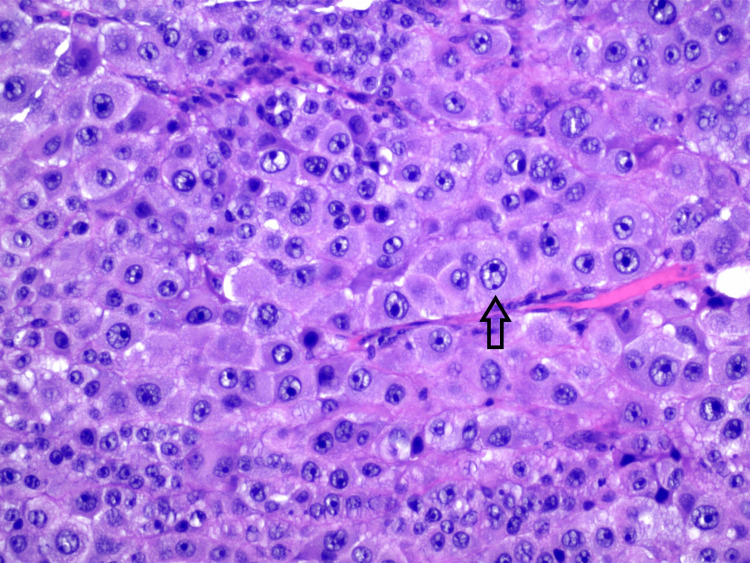
Hematoxylin and eosin stain (H&E), 40x. Cells with large pleomorphic nuclei, prominent nucleoli, and abundant amphophilic cytoplasm (arrow). No pigment, no squamous differentiation, and no glandular differentiation

**Figure 7:**
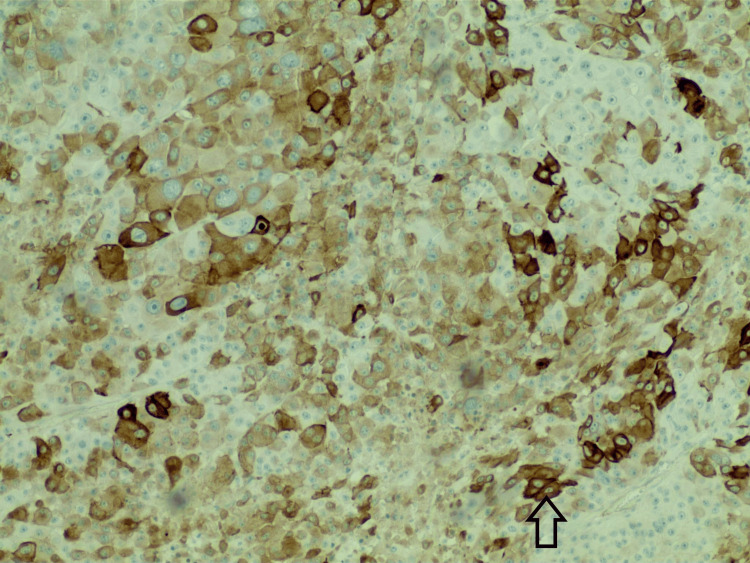
MART1 immunohistochemical stain. 10x. Cytoplasmic staining (arrow). Supports diagnosis of melanoma

**Figure 8:**
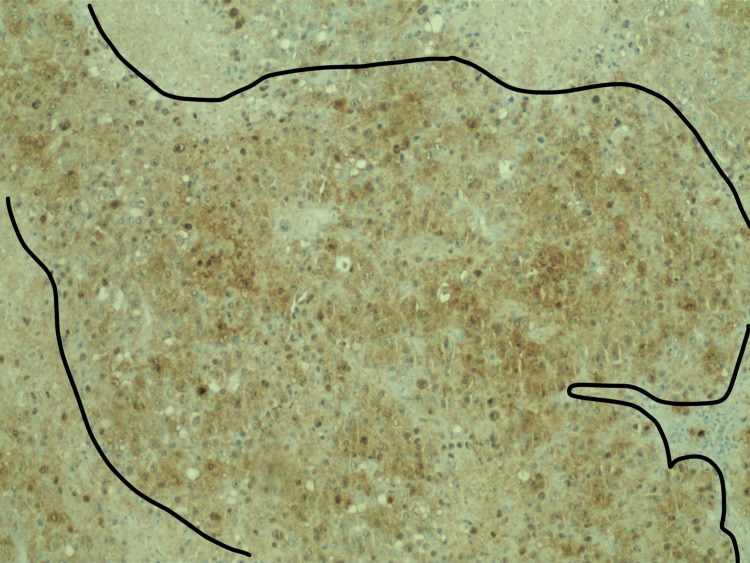
S100 immunohistochemical stain, 10x. Predominantly cytoplasmic staining, but some nuclear staining (within marked borders). Supports diagnosis of melanoma

**Figure 9:**
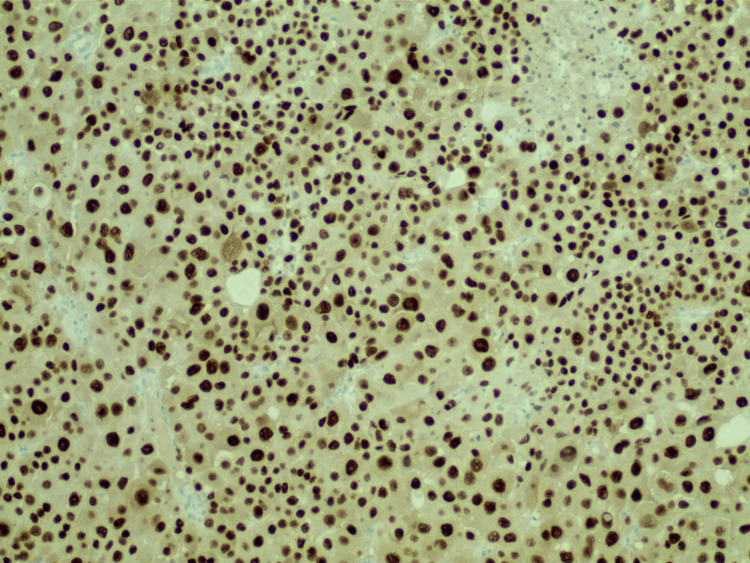
SOX10 immunohistochemical stain, 10x. Nuclear stain. Supports diagnosis of melanoma
